# Elevated levels of matrix metalloproteinases reflect severity and extent of disease in tuberculosis-diabetes co-morbidity and are predominantly reversed following standard anti-tuberculosis or metformin treatment

**DOI:** 10.1186/s12879-018-3246-y

**Published:** 2018-07-25

**Authors:** Nathella P. Kumar, Kadar Moideen, Vijay Viswanathan, Basavaradhya S. Shruthi, Shanmugam Sivakumar, Pradeep A. Menon, Hardy Kornfeld, Subash Babu

**Affiliations:** 1 National Institutes of Health–NIH-NIRT-ICER, National Institute for Research in Tuberculosis, International Center for Excellence in Research, # 1 Mayor Sathyamoothy Road, Chetpet, Chennai, India; 2 Prof. M. Viswanathan Diabetes Research Center, Chennai, India; 3 National Institute for Research in Tuberculosis, Chennai, India; 4 University of Massachusetts Medical School, Worcester, MA USA; 5 LPD, NIAID, NIH, MD, Bethesda, USA

**Keywords:** *Mycobacterium tuberculosis*, Diabetes mellitus, Matrix metalloproteinases

## Abstract

**Background:**

Matrix metalloproteinases (MMPs) are considered to be key mediators of tuberculosis (TB) pathology but their role in tuberculosis – diabetes comorbidity (TB-DM) is not well understood.

**Methods:**

To study the association of MMP levels with severity and extent of disease as well as bacterial burden in TB-DM, we examined the systemic levels of MMP-1, − 2, − 3, − 7, − 8, − 9, − 10, − 12 and − 13 in individuals with TB-DM and compared them to those with TB alone (TB) or healthy controls (HC).

**Results:**

Circulating levels of MMP-1, − 2, − 3, − 7, − 10 and − 12 were significantly higher in TB-DM compared to both TB and HC and MMP -13 levels were higher in comparison to HC alone. To understand the effect of standard anti-tuberculosis therapy (ATT) on these MMP levels in TB-DM, we measured the levels of MMPs at the end of treatment (post-treatment). Our findings indicate that ATT is associated with a significant reduction in the levels of MMP-1, − 2, − 3, − 8 and − 13 post-treatment. Moreover, the levels of MMP-1, − 2, − 3, − 9 and − 12 were significantly higher in TB-DM individuals with cavitary disease and/or bilateral disease at baseline but not post-treatment. Similarly, the levels of MMP -1, − 2, − 3 and − 8 exhibited a significant positive relationship with bacterial burden and HbA1c levels at baseline but not post-treatment. Within the TB-DM group, those known to be diabetic before incident TB (KDM) exhibited significantly higher levels of MMP-1, − 2, − 10 and − 12 at baseline and of MMP-1 and -3 post-treatment compared to those newly diagnosed with DM (NDM). Finally, KDM individuals on metformin treatment exhibited significantly lower levels of MMP-1, − 2, − 3, − 7, − 9 and − 12 at baseline and of MMP-7 post-treatment.

**Conclusions:**

Our data demonstrate that systemic MMP levels reflect baseline disease severity and extent in TB-DM, differentiate KDM from NDM and are modulated by ATT and metformin therapy.

**Electronic supplementary material:**

The online version of this article (10.1186/s12879-018-3246-y) contains supplementary material, which is available to authorized users.

## Background

Matrix metalloproteinases cover a large family of extracellular enzymes that share common structural features, predominantly those regions implicated in proteolytic activity [1]. Twenty-eight different vertebrate MMPs have been cloned to date and additional members continue to be identified. MMPs play a substantial role in diverse biological functions involving many characteristics of the immune response [2, 3]. These MMPs can also function on pro-inflammatory cytokines, chemokines and other proteins to regulate various aspects of inflammation and immunity [3].

Previous studies have demonstrated that MMP levels are increased in human TB and correlate strongly with clinical and radiological markers of lung tissue destruction [4]. In spite of crucial role of MMPs in lung matrix destruction in human TB, the principal mechanisms resulting in tissue damage have not been defined. In addition, we have shown that MMP-1, − 7 and − 8 plasma levels were significantly elevated in children with pulmonary TB [5]. We have also shown that MMP-1 is an important biomarker for the discrimination of TB-DM from TB [6]. The anti-diabetic drug metformin was previously described to exert anti-mycobacterial activity in vitro and in vivo and treatment with metformin has been shown to reverse the heightened mortality linked with TB-DM [7, 8]. However, a comprehensive examination of the association of MMPs with TB-DM and their relationship to disease pathology or bacterial burden has not been carried out. Similarly, the relationship of MMPs with TB individuals with KDM or NDM and among TB-KDM individuals with or without metformin use has never been examined. Since TB-DM is characterized by increased immune pathology compared to TB alone [6, 9], we postulated that one potential mechanism could be a systemic expansion in the levels of MMPs in TB-DM individuals.

To address these gaps in knowledge, we examined the association of the systemic levels of MMP-1, − 2, − 3, − 7, − 8, − 9, − 10, − 12 and − 13 in TB-DM individuals and compared them with TB and HC individuals. We demonstrate elevated levels of MMPs in association with TB-DM. We also determine the association of MMPs with the extent and severity of lung disease and with bacterial burden at baseline, indicating that MMPs are a reflection of immune pathology in TB-DM. In addition, we show the reversability of these findings following ATT. Finally, we also demonstrate increase in MMPs in KDM compared to NDM individuals and decrease in MMPs in those using metformin therapy.

## Methods

### Ethics statement

The Ethical committees of Prof. M. Viswanathan Diabetes Research Center and National Institute for Research in Tuberculosis have approved the study. Informed written consent was obtained from all participants.

### Study population

Plasma samples were collected from 64 individuals with active pulmonary TB with diabetes mellitus (TB-DM) and 24 individuals with active pulmonary TB (TB) and 24 healthy control individuals, enrolled in Chennai, India. Pulmonary TB was diagnosed based on smear and culture positivity for *Mycobacterium tuberculosis* (*M.tb*). To define cavitary disease as well as unilateral versus bilateral lung involvement, chest X-rays were done for all the enrolled TB patients. Smear grades were used to determine bacterial burden and classified as 1+, 2+ and 3+. During the time of recruitment, all active TB cases had no record of prior TB disease or anti-TB treatment (ATT). Glycemic status (DM or normoglycemia) was diagnosed on the basis of oral glucose tolerance test and/or glycated hemoglobin (HbA1c) levels (for known diabetics), according to the WHO criteria. Amongst the 64 TB-DM individuals, 32 were known diabetics (KDM) and 32 were newly diagnosed diabetics (NDM). Amongst the KDM individuals, 16 were on metformin containing anti-diabetic medication and 16 were not. Healthy control individuals were asymptomatic, had normal chest x-rays and were non-diabetic. All individuals were BCG vaccinated, HIV negative and had normal body mass index. The study groups were similar with regard to age and gender and the baseline characteristics of the study participants are shown in Table 1. Standard ATT was administered to TB-DM individuals using the directly observed treatment, short course (DOTS) strategy. At 6 months following ATT initiation, fresh plasma samples were obtained. All TB-DM individuals were culture negative at the end of ATT.

**Table 1:** Demographics of the study groups and biochemical parameters in TB-DM TB and HC

Study Demographics	TB-DM	TB	HC	*p* Value
No. of subjects recruited	64	24	24	–No significance
Gender (Male / Female)	44/20	17/7	14/10	–No significance
Median Age (Range)	52 (31–70)	43 (30–67)	35(27–62)	–No significance
Median Height, cm	159 (129–176)	164 (121–181)	162 (125–190)	–No significance
Median Weight, kg	49 (31–64)	44 (30–90)	55 (45–90)	–No significance
Body mass index kg/m^2^	19.3 (13.2–32.6)	17.2 (12.2–21.2)	22.3 (18.2–24.7)	–No significance
Smear Grade: 0/1+/2+/3+	0/22/24/18	0/9/9/6	NA	–No significance
Fasting Blood Glucose, mg/dL	158 (109–427)	93 (73–103)	88 (75–105)	***p*** **< 0.0001**
Post Prandial Glucose, mg/dL	220 (183–448)	112 (80–129)	110 (78–120)	***p*** **< 0.0001**
Glycated hemoglobin level, %	10.3 (7.3–15.6)	5.6 (5.0–5.8)	5.5 (5.0–5.7)	***p*** **< 0.0001**

The values represent the geometric mean (and the 95% confidence intervals) except for age where the median (and the range) are depicted. *P* values were calculated using the Kruskal-Wallis test with Dunn’s post-hoc for multiple comparisons

*P* values captured in bold are statistically significant

### Elisa

Circulating levels of MMP-1, MMP-2, MMP-3, MMP-7, MMP-8, MMP-9, MMP-10, MMP-12 and MMP-13 were determined using a multiplex enzyme-linked immunosorbent assay system (Bio-Rad Laboratories, Inc) in plasma samples. The lowest detection limits were as follows: MMP-1, 115.8 pg/mL; MMP-2, 809 pg/mL; MMP-3, 199.2 pg/mL; MMP-7, 27.7 pg/mL; MMP-8, 31.7 pg/mL; MMP-9, 257.5 pg/mL; MMP-10, 78.4 pg/mL; MMP-12, 18.5 pg/mL; MMP-13, 32.9 pg/mL.

### Statistical analysis

Geometric means (GM) were used for measurements of central tendency. Statistically significant differences between three groups were examined using the Kruskal-Wallis test with Dunn's post-hoc test. Statistically significant differences between two groups were examined using the Mann Whitney test with Holm’s correction for multiple comparisons. Wilcoxon signed rank test was used to compare MMP concentrations before and after ATT. Linear trend post-test was used to compare MMP concentrations with smear grades (reflecting bacterial burden) and Spearman rank correlation was used to compare MMP concentrations with HbA1c levels. Analyses were performed using GraphPad PRISM Version 6.01.

## Results

### Study population characteristics

The baseline characteristics comprising demographic and biochemical features of the study population are shown in Table 1. TB-DM individuals had significantly increased levels of fasting and post-prandial glucose as well as glycated haemoglobin compared TB and HC individuals. No significant differences were observed in age, sex, smear or culture grades at baseline between the TB-DM and TB groups (Table 1).

### Heightened levels of circulating MMPs in TB-DM and reversal following ATT

**Fig. 1:**
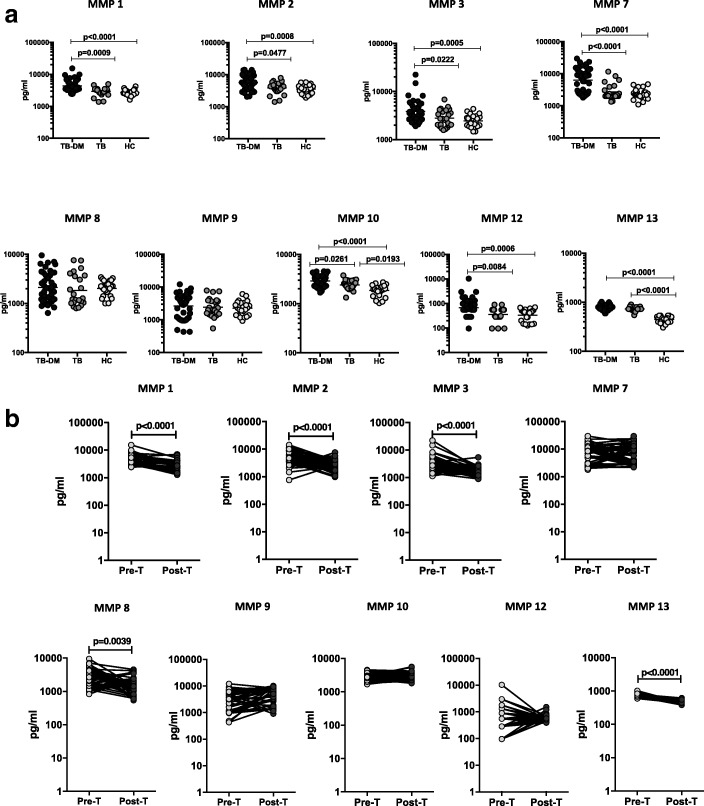
Elevated circulating levels of MMPs in TB-DM individuals. (**a**) The plasma levels of MMPs were measured in TB-DM (*n* = 64), TB (*n* = 24) and HC (n = 24) individuals at baseline. The data are represented as scatter plots with each circle representing a single individual. *P* values were calculated using the Kruskal-Wallis test with Dunn’s post-hoc for multiple comparisons (**b**) The plasma levels of MMPs were measured in TB-DM individuals at baseline (pre-T) and at 6 months of ATT (post-T). The data are presented as line graphs with each line representing a single individual. *P* values were calculated using the Wilcoxon signed rank test

As MMPs are linked with inflammation and tissue damage as well as matrix remodelling in TB, we wanted to examine the systemic levels of circulating MMPs in TB-DM, TB and HC individuals. To this end, we estimated the plasma levels of MMP-1, − 2, − 3, − 7, − 8, − 9, − 10, − 12 and − 13 by multi-plex ELISA in TB-DM (*n* = 64), TB (*n* = 24) and HC (n = 24) individuals (Fig. 1). As shown in Fig. 1a, systemic levels of MMP-1, − 2, − 3, − 7, − 10, − 12 and − 13 (Additional file 1: Table S1) were significantly higher in TB-DM compared to TB and HC individuals at baseline. To study the effect of ATT on MMP levels in TB-DM, we measured the levels of MMPs in TB-DM before (pre-T) and at the end of ATT (post-T). As shown in Fig. 1b, systemic levels of MMP-1, − 2, − 3, − 8 and − 13 (Additional file 2: Table S2) were significantly diminished in TB-DM at 6 months post-T in comparison to pre-T levels. Thus, TB-DM is associated with heightened systemic levels of circulating MMPs at baseline and reversal following standard ATT.

### Circulating MMPs are markers of disease severity and extent in TB-DM

**Fig. 2:**
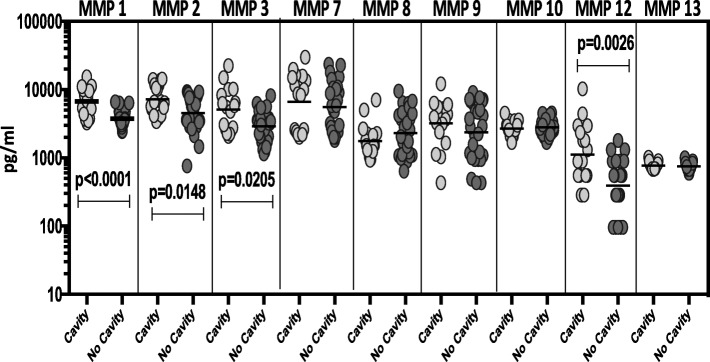
Elevated circulating levels of MMPs in cavitary disease in TB-DM individuals. The plasma levels of MMPs were measured in TB-DM individuals with cavitary versus non-cavitary disease at baseline. The data are represented as scatter plots with each circle representing a single individual. *P* values were calculated using the Mann-Whitney test with Holm’s correction for multiple comparisons

To elucidate the association between the systemic levels of circulating MMPs and disease severity in TB-DM, we measured the plasma levels of MMPs in TB-DM individuals with cavitary versus non-cavitary disease. As shown in Fig. 2, the circulating levels of MMP-1, − 2, − 3 and − 12 (Additional file 3: Table S3) were increased in TB-DM individuals with cavitary disease compared to those without. However, upon completion of ATT and the consequent healing of cavitary lesions, the circulating MMP levels exhibited no significant difference between the individuals who presented initially with cavity compared to those did not.

**Fig. 3:**
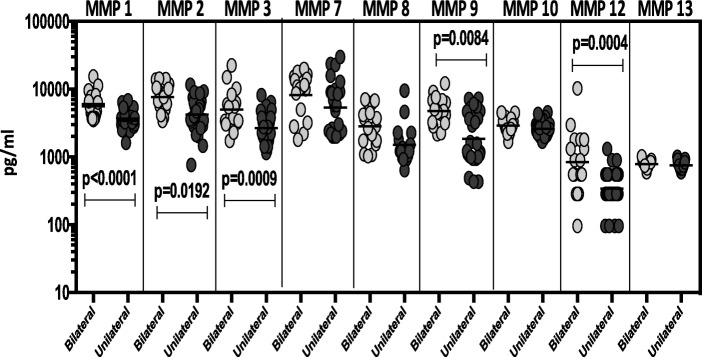
Elevated circulating levels of MMPs in bilateral disease in TB-DM individuals. The plasma levels of MMPs were measured in TB-DM individuals with bilateral versus unilateral disease at baseline. The data are represented as scatter plots with each circle representing a single individual. *P* values were calculated using the Mann-Whitney test with Holm’s correction for multiple comparisons

To elucidate the association between the systemic levels of circulating MMPs and the radiographic extent of disease in TB-DM, we measured the plasma levels of MMPs in TB-DM individuals with bilateral versus unilateral disease at baseline and post-treatment. As shown in Fig. 3, the circulating levels of MMP-1, − 2, − 3, − 9 and − 12 (Additional file 3: Table S3) were significantly increased in TB-DM individuals with bilateral disease compared to those with unilateral disease. However, upon completion of ATT and the consequent healing, the circulating MMP levels exhibited no significant difference between the individuals who presented initially with bilateral disease compared to those did not. Thus, disease severity and extent in TB-DM is linked with increased systemic levels of circulating MMPs at baseline but not after completion of TB treatment.

### Correlation of circulating MMPs with estimated bacterial burden

**Fig. 4:**
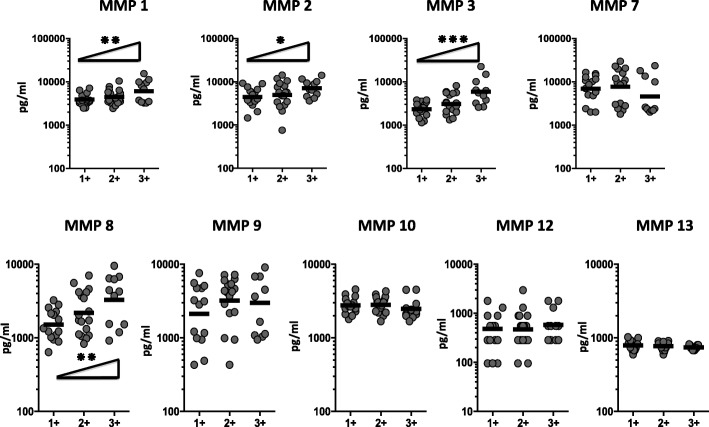
Circulating MMPs are marker of bacterial burden in TB-DM. The relationship between the plasma levels of MMPs and smear grades as estimated by sputum smears was examined in TB-DM individuals at baseline. The data are represented as scatter plots with each circle representing a single individual. P values were calculated using the Linear trend post – test

To elucidate the association of circulating MMPs and bacterial burden, we done a correlation of the circulating levels of MMPs in TB-DM individuals with smear grades. As shown in Fig. 4, MMP-1, − 2, − 3 and − 8 displayed a significant positive correlation with smear grades in TB-DM individuals, showing a positive association of these factors with bacterial burden. However, upon completion of ATT the circulating MMP levels revealed no significant correlation in TB-DM individuals with smear grades. Thus, bacterial burden in TB-DM are linked with heightened systemic levels of circulating MMPs at baseline but not after successful completion of TB treatment.

### Circulating MMPs exhibit a positive relationship with HbA1c in TB-DM

**Fig. 5:**
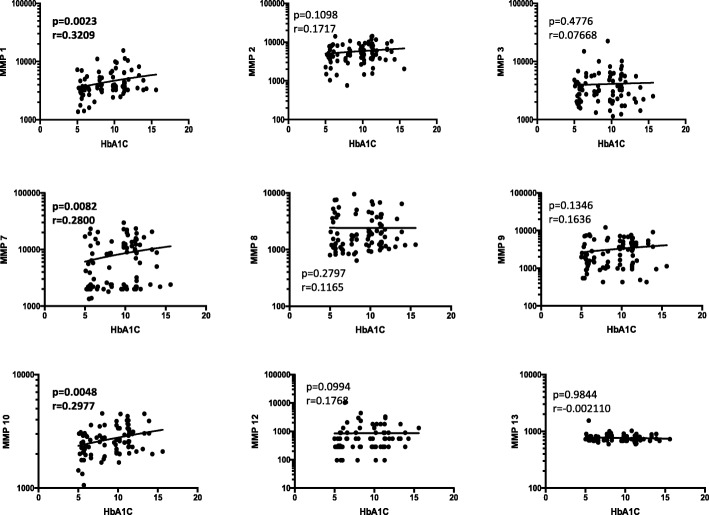
Significant correlation between circulating levels of MMPs and glycemic parameters in all TB-DM individuals. The relationship between the plasma levels of MMPs and HbA1c levels was examined in all PTB individuals with and without DM at baseline. The data are represented as scatter plots with each circle representing a single individual. P values were calculated using the Spearman Rank Correlation

To elucidate the association between systemic levels of circulating MMPs and glycemic control in TB-DM, we determined the relationship between the circulating levels of MMPs in TB-DM with HbA1c levels (Fig. 5). As shown, the systemic levels of MMP-1 exhibited a significant positive relationship, wear as MMP-7 and MMP-10 displayed a borderline positive correlation with HbA1c levels in TB-DM at baseline, showing a significant association of these factors with poor glycemic control. Similarly, only the systemic levels of MMP-10 exhibited a significant positive relationship with HbA1c levels at post-treatment, indicating a significant reversal of the association of MMPs with poor glycemic control at the end of ATT.

### Known DM is associated with increased circulating levels of MMPs during pre and post treatment conditions

**Fig. 6:**
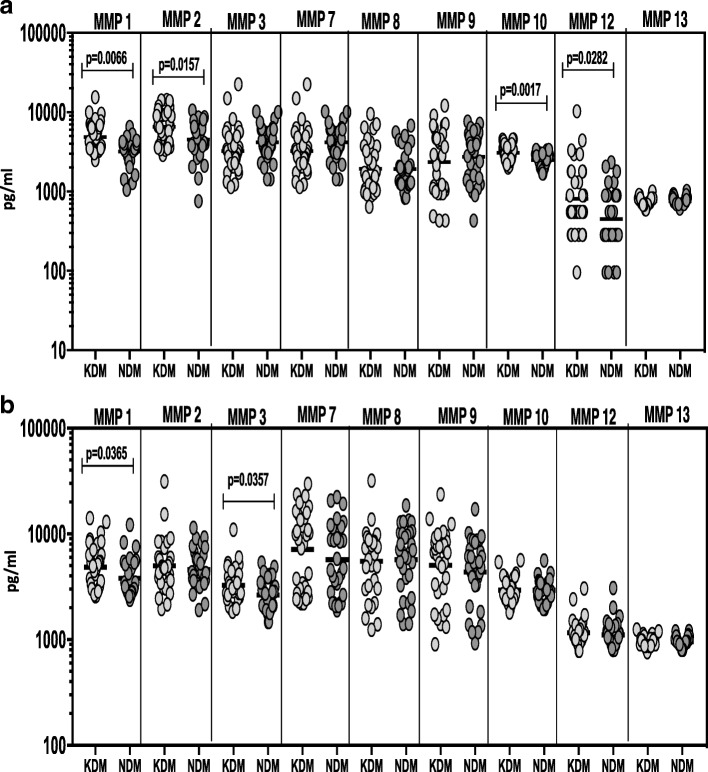
Elevated circulating levels of MMPs in KDM individuals. (**a**) The plasma levels of MMPs were measured in TB-DM individuals with known diabetes. (KDM) versus newly diagnosed diabetes (NDM) at baseline. (**b**) The plasma levels of MMPs were measured in TB-DM individuals with known diabetes (KDM) versus newly diagnosed diabetes (NDM) at 6 months of ATT. The data are represented as scatter plots with each circle representing a single individual. P values were calculated using the Mann-Whitney test with Holm’s correction for multiple comparisons

To determine whether MMP levels differ between known diabetic individuals (KDM) and newly diagnosed diabetes (NDM) in TB-DM individuals, we measured the circulating levels of MMPs in KDM (*n* = 32) and NDM (n = 32) individuals (Fig. 6). As shown in Fig. 6a, systemic levels of MMP-1, − 2, − 10 and − 12 (Additional file 4: Table S4) were significantly higher in KDM compared to NDM individuals at baseline. As shown in Fig. 6b, systemic levels of MMP-1 and MMP-3 (Additional file 4: Table S4) were significantly higher in KDM compared to NDM individuals upon completion of ATT. Thus, KDM is associated with elevated systemic levels of circulating MMPs at baseline as well as following standard ATT.

### Metformin treatment associated with diminished circulating MMPs

**Fig. 7:**
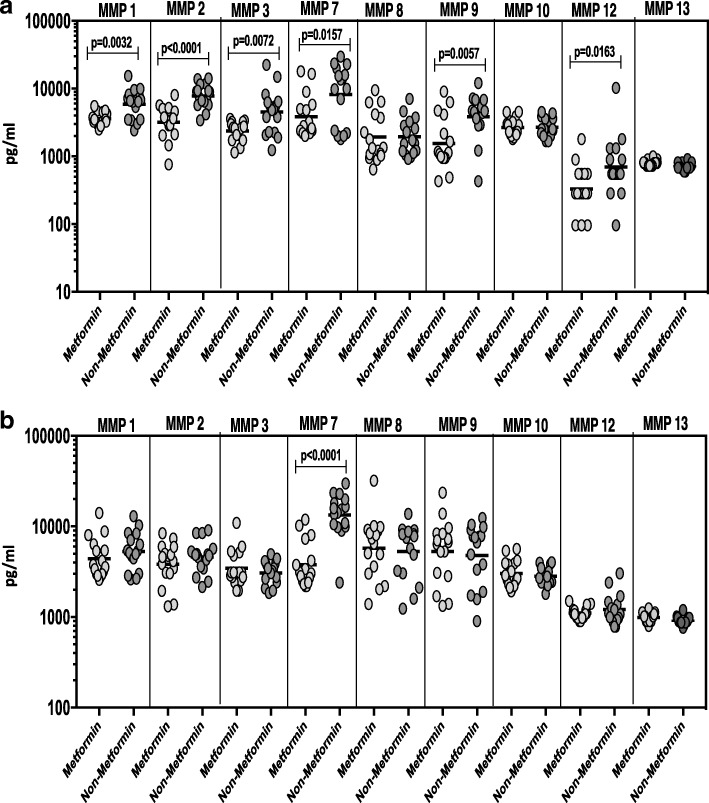
Diminished circulating levels of MMPs in KDM individuals on metformin treatment. (**a**) MMPs were measured in KDM individuals on metformin treatment versus no metformin treatment at baseline. (**b**) The plasma levels of MMPs were measured in KDM individuals on metformin treatment versus no metformin treatment at 6 months of ATT. The data are represented as scatter plots with each circle representing a single individual. P values were calculated using the Mann-Whitney test with Holm’s correction for multiple comparisons

Since the anti-diabetic drug metformin is associated with protection against mortality in TB-DM, we wanted to examine the systemic levels of circulating MMPs in KDM individuals on metformin treatment (*n* = 16) compared to those on non-metformin regimens (n = 16). No significant differences were observed in HbA1c levels between KDM individuals on metformin compared to KDM individuals not on metformin. As shown in Fig. 7a, systemic levels of MMP-1, − 2, − 3, − 7, − 9 and − 12 (Additional file 5: Table S5) were significantly diminished in KDM individuals on metformin compared to KDM individuals not on metformin. As shown in Fig. 7B, MMP-7 (Additional file 5: Table S5) alone was significantly diminished in KDM individuals on metformin compared to KDM individuals not on metformin upon completion of ATT. Thus, metformin therapy in KDM individuals is associated with diminished systemic levels of circulating MMPs.

## Discussion

The increased severity of TB disease in the face of DM comorbidity has a major negative impression on public health, specifically in the countries where both diseases are vastly endemic. There is also strong confirmation that DM contributes significantly to TB incidence, which in is turn linked with poor TB treatment outcomes [10, 11]. The immunological basis for enhanced susceptibility in TB-DM comorbidity is relatively unknown, although recently published data advocates that innate and adaptive immune responses might be affected [11–13]. MMPs are a family of zinc-dependent proteases consisting of two conserved domains, a prodomain and a catalytic domain [14]. Collectively MMPs can successfully degrade all elements of the extracellular matrix, including collagens, laminin, fibrillar, vitronectin, and proteoglycans [14]. After *M.tb* infection, the local cellular and adjacent tissues are remodelled to accelerate leukocyte infiltration and the initiation of granuloma formation and previously published cellular and animal studies report that MMPs play an important role in the cellular recruitment, tissue remodelling, and destruction [15]. MMP activity has been involved in driving TB pathology, and it has critical immunological and pathological roles. Although MMPs have been reviewed broadly in other destructive pulmonary pathologies [16], their role in TB disease and pathogenesis is still being explored.

Multiple MMPs are up-regulated in human TB and it has been shown that plasma concentrations of MMP-1, − 7 and − 8 were increased in TB patients compared to controls [17–19]. Similarly, MMP-1, − 2, − 3, − 8 and − 9 are elevated in sputum samples of TB patients compared to healthy volunteers [20]. Published studies from TB-HIV coinfection reported that in sputum, multiple MMPs are elevated in TB patients compared to controls. However authors reported that TB (HIV+) displayed lower median sputum MMP-1, − 2, − 3, and − 9 concentrations compared to TB (HIV−). Together, this data supports a role for sputum MMPs involved in pulmonary TB driven matrix degradation [21]. MMP-1, − 2, − 8 and − 9 were found to be elevated in pleural fluid of patients with TB compared to pleural fluid of non-TB pleuritis [22–24]. MMP-9 concentrations are increased in the cerebrospinal fluid of patients with TB meningitis [25] and correlate with extent of neurological compromise [26]. We previously reported that that MMP-1, − 7 and − 8 plasma levels were significantly elevated in children with pulmonary TB compared to healthy controls [5].

In agreement with previous reports that systemic levels of MMPs increase significantly with the severity of TB disease and that clinical severity of TB is increased by comorbid DM, our current study revealed that TB-DM individuals exhibit significantly higher systemic levels of MMP-1, − 2, − 3, − 7, − 10, − 12 and − 13 compared to TB and healthy individuals. Our data also revealed a significant association of MMP levels with the severity of TB disease (as estimated by the bilateral and cavitary disease) and increasing bacterial burden only at baseline, indicating that comorbid DM amplifies this response, which might mirror increased bacterial load and/or a specific perturbation of immune function. Thus, MMPs appear to be linked with pathology and bacterial burden in TB-DM. Of additional interest are the findings that MMP levels are positively correlated with HbA1c, indicating an association with poor glycemic control which drives diabetic complications in all tissues. [27, 28]. The published data reports that elevated MMP-8 and -9 levels were directly connected with neutrophil markers, with MMP-8 expressing neutrophils placed in the wall of TB cavities, which in turn imply a role of neutrophils in driving tissue destruction and cavitation in TB [4]. Similarly, TB patients with extensive tissue destructive disease on the chest X-ray were shown to have augmented sputum MMP-1 levels compared with those with less tissue damage [29]. The heightened levels of multiple MMPs suggest that a number of proteases may be a factor for tissue destruction and cavitation in TB, but their relative significance is presently unknown. Our results, therefore, imply that MMP inhibition might be uniquely useful in host directed therapy (HDT) in TB-DM patients.

After completion of anti TB treatment, matrix-degrading phenotype solves quickly in patients with fully drug-sensitive pulmonary TB and the levels of MMP-1, − 3 and − 8 concentrations in sputum decline markedly in the first 2 weeks of anti-TB treatment [20]. Consistent with that observation, we observed a significant reversal of MMP levels in most TB-DM patients at the completion of TB treatment. With radiographic and microbiological improvement, MMP levels fall in most TB-DM patients and become indistinguishable from TB alone. Thus, the pattern of MMP elevation and correlation with radiographic features and sputum status suggest that DM is associated with increased TB disease severity that might be mediated in part by MMP activities, and this condition is treatment-responsive. While a few previous reports have described post-treatment effects on MMPs, this study is the first to our knowledge to extensively characterize the effect of ATT on MMP levels in TB disease.

A previous study from our group reported that there was a bimodal distribution of baseline HbA1c between KDM and NDM individuals in the EDOTS study cohort, with significantly higher baseline A1c in the KDM group [30]. Our current study adds to this apparent heterogeneity in the presentation of TB-DM comorbidity. We found that circulating MMPs were significantly enhanced in KDM compared to NDM groups at baseline and after completion anti-TB treatment, reflecting the increased severity of TB disease in KDM individuals. The most frequently-prescribed anti-diabetic agent metformin has drawn attention as a potential adjunctive, host-directed therapy (HDT) for TB independent of its glucose-lowering activity [7, 31, 32]. Studies from murine models reported that metformin treatment was found to reduce *M.tb* growth and improve lung pathology. The use of metformin-containing DM treatment regimens also is associated with reduced risk for TB progression and decreased mortality and lung cavitation in active TB patients [7, 8]. Our findings provide new evidence for a host – directed role for metformin in that individuals on metformin treatment exhibited diminished systemic MMP levels, suggesting a host – protective effect of metformin in TB-DM with possible implications for its use in TB without DM.

## Conclusion

Our data on MMPs overall suggest that upregulation of MMPs is a typical characteristic of TB-DM co-morbidity. Our data also add to the growing list of evidence indicating heightened immune activation in this immune-metabolic nexus. MMPs appear to act as reliable and reproducible biomarkers for therapeutic monitoring of TB-DM disease. Finally, our study reinforces the necessity of aggressively controlling the dysregulated glucose metabolism and inflammatory milieu that characterized TB-DM to minimize inflammatory pathology and possibly poor outcomes in TB-DM co-morbidity. Therefore, our findings demonstrate that systemic MMP levels reflect baseline disease severity and extent in TB-DM, differentiate KDM from NDM, and are modulated by ATT and metformin therapy.

## Additional files

Additional file 1: **Table S1.** The plasma levels of MMPs were measured in TB-DM (*n* = 64), TB (*n* = 24) and HC (n = 24). (DOCX 14 kb)

Additional file 2: **Table S2.** The plasma levels of MMPs were measured in TB-DM individuals at baseline (pre-T) and at 6 months of ATT (post-T). (DOCX 13 kb)

Additional file 3: **Table S3.** The plasma levels of MMPs were measured in TB-DM individuals cavitary versus non-cavitary disease and bilateral versus unilateral disease. (DOCX 13 kb)

Additional file 4: **Table S4.** The plasma levels of MMPs were measured in TB-DM individuals with known diabetes (KDM) versus newly diagnosed diabetes (NDM) at baseline and 6 months of ATT. (DOCX 13 kb)

Additional file 5: **Table S5.** MMPs were measured in KDM individuals on metformin treatment versus no metformin treatment at baseline and 6 months of ATT. (DOCX 13 kb)

## Abbreviations

ATT: anti-TB treatment
DM: diabetes mellitus
DOTS: directly observed treatment, short course
GM: Geometric means
HbA1c: glycated hemoglobin
HC: healthy controls
HDT: Host directed therapy
KDM: diabetic before incident TB
*M.tb*: *Mycobacterium tuberculosis*
MMPs: Matrix metalloproteinases
NDM: newly diagnosed with DM
TB: tuberculosis
