# Supplementary material for: Elevated levels of matrix metalloproteinases reflect severity and extent of disease in tuberculosis-diabetes co-morbidity and are predominantly reversed following standard anti-tuberculosis or metformin treatment
Source: BMC Infect Dis. 2018 Jul 25;18:345. doi: 10.1186/s12879-018-3246-y (PMC6060542; doi:10.1186/s12879-018-3246-y)
Supplement: Supplementary file 1 — Table S1. The plasma levels of MMPs were measured in TB-DM (n = 64), TB (n = 24) and HC (n = 24). (DOCX 14 kb) [file 12879_2018_3246_MOESM1_ESM.docx]

Additional file 1: Table S1 The plasma levels of MMPs were measured in TB-DM (n=64), TB (n=24) and HC (n=24)

| **GeoMean** | **TB-DM** | **TB** | **HC** |
| --- | --- | --- | --- |
| **MMP-1 (pg/ml)** | 4332 | 2929 | 2713 |
| **MMP-2 (pg/ml)** | 5614 | 3873 | 3534 |
| **MMP-3 (pg/ml)** | 3832 | 2781 | 2452 |
| **MMP-7 (pg/ml)** | 6304 | 2705 | 2344 |
| **MMP-10 (pg/ml)** | 2908 | 2391 | 1857 |
| **MMP-12 (pg/ml)** | 671.4 | 353.1 | 334.7 |
| **MMP-13 (pg/ml)** | 764.5 | 733.4 | 447.6 |
