# Supplementary material for: Elevated levels of matrix metalloproteinases reflect severity and extent of disease in tuberculosis-diabetes co-morbidity and are predominantly reversed following standard anti-tuberculosis or metformin treatment
Source: BMC Infect Dis. 2018 Jul 25;18:345. doi: 10.1186/s12879-018-3246-y (PMC6060542; doi:10.1186/s12879-018-3246-y)
Supplement: Supplementary file 2 — Table S2. The plasma levels of MMPs were measured in TB-DM individuals at baseline (pre-T) and at 6 months of ATT (post-T). (DOCX 13 kb) [file 12879_2018_3246_MOESM2_ESM.docx]

Additional file 2: Table S2 The plasma levels of MMPs were measured in TB-DM individuals at baseline (pre-T) and at 6 months of ATT (post-T)

| **GeoMean** | **Pre-T** | **Post-T** |
| --- | --- | --- |
| **MMP-1 (pg/ml)** | 4332 | 2252 |
| **MMP-2 (pg/ml)** | 5614 | 2433 |
| **MMP-3 (pg/ml)** | 3832 | 1612 |
| **MMP-8 (pg/ml)** | 2474 | 1528 |
| **MMP-13 (pg/ml)** | 764.5 | 481 |
