# Supplementary material for: Elevated levels of matrix metalloproteinases reflect severity and extent of disease in tuberculosis-diabetes co-morbidity and are predominantly reversed following standard anti-tuberculosis or metformin treatment
Source: BMC Infect Dis. 2018 Jul 25;18:345. doi: 10.1186/s12879-018-3246-y (PMC6060542; doi:10.1186/s12879-018-3246-y)
Supplement: Supplementary file 3 — Table S3. The plasma levels of MMPs were measured in TB-DM individuals cavitary versus non-cavitary disease and bilateral versus unilateral disease. (DOCX 13 kb) [file 12879_2018_3246_MOESM3_ESM.docx]

Additional file 3: Table S3 The plasma levels of MMPs were measured in TB-DM individuals cavitary versus non-cavitary disease and bilateral versus unilateral disease.

| **GeoMean** | **Cavity** | **No Cavity** |
| --- | --- | --- |
| **MMP-1 (pg/ml)** | 6713 | 3730 |
| **MMP-2 (pg/ml)** | 7243 | 4911 |
| **MMP-3 (pg/ml)** | 5100 | 3278 |
| **MMP-12 (pg/ml)** | 1119 | 485 |
| **GeoMean** | **Bilateral** | **Unilateral** |
| **MMP-1 (pg/ml)** | 5927 | 3627 |
| **MMP-2 (pg/ml)** | 6794 | 4767 |
| **MMP-3 (pg/ml)** | 5069 | 3006 |
| **MMP-9 (pg/ml)** | 3820 | 2029 |
| **MMP-12 (pg/ml)** | 1043 | 437 |
