# Supplementary material for: Elevated levels of matrix metalloproteinases reflect severity and extent of disease in tuberculosis-diabetes co-morbidity and are predominantly reversed following standard anti-tuberculosis or metformin treatment
Source: BMC Infect Dis. 2018 Jul 25;18:345. doi: 10.1186/s12879-018-3246-y (PMC6060542; doi:10.1186/s12879-018-3246-y)
Supplement: Supplementary file 4 — Table S4. The plasma levels of MMPs were measured in TB-DM individuals with known diabetes (KDM) versus newly diagnosed diabetes (NDM) at baseline and 6 months of ATT. (DOCX 13 kb) [file 12879_2018_3246_MOESM4_ESM.docx]

Additional file 4: Table S4 The plasma levels of MMPs were measured in TB-DM individuals with known diabetes. (KDM) versus newly diagnosed diabetes (NDM) at baseline and 6 months of ATT

| **GeoMean**  **(Baseline)** | **KDM** | **NDM** |
| --- | --- | --- |
| **MMP-1 (pg/ml)** | 4830 | 3210 |
| **MMP-2 (pg/ml)** | 6539 | 4533 |
| **MMP-10 (pg/ml)** | 3071 | 2487 |
| **GeoMean**  **(Post treatment)** | **KDM** | **NDM** |
| **MMP-1 (pg/ml)** | 4322 | 3732 |
| **MMP-3 (pg/ml)** | 3258 | 2640 |
