# Supplementary material for: Elevated levels of matrix metalloproteinases reflect severity and extent of disease in tuberculosis-diabetes co-morbidity and are predominantly reversed following standard anti-tuberculosis or metformin treatment
Source: BMC Infect Dis. 2018 Jul 25;18:345. doi: 10.1186/s12879-018-3246-y (PMC6060542; doi:10.1186/s12879-018-3246-y)
Supplement: Supplementary file 5 — Table S5. MMPs were measured in KDM individuals on metformin treatment versus no metformin treatment at baseline and 6 months of ATT. (DOCX 13 kb) [file 12879_2018_3246_MOESM5_ESM.docx]

Additional file 5: Table S5 MMPs were measured in KDM individuals on metformin treatment versus no metformin treatment at baseline and 6 months of ATT

| **GeoMean**  **(Baseline)** | **KDM** | **NDM** |
| --- | --- | --- |
| **MMP-1 (pg/ml)** | 3611 | 5879 |
| **MMP-2 (pg/ml)** | 3206 | 7796 |
| **MMP-3 (pg/ml)** | 2358 | 4511 |
| **MMP-7 (pg/ml)** | 3872 | 8161 |
| **MMP-9 (pg/ml)** | 1546 | 3831 |
| **MMP-12 (pg/ml)** | 330 | 697 |
| **GeoMean**  **(Post treatment)** | **KDM** | **NDM** |
| **MMP-7 (pg/ml)** | 3798 | 8797 |
